# Exploiting Lipid and Polymer Nanocarriers to Improve the Anticancer Sonodynamic Activity of Chlorophyll

**DOI:** 10.3390/pharmaceutics12070605

**Published:** 2020-06-30

**Authors:** Federica Bosca, Federica Foglietta, Alberto Gimenez, Roberto Canaparo, Giovanni Durando, Ilaria Andreana, Alessandro Barge, Elena Peira, Silvia Arpicco, Loredana Serpe, Barbara Stella

**Affiliations:** 1 Department of Drug Science and Technology, University of Torino, 10125 Torino, Italy; federica.bosca@unito.it (F.B.); federica.foglietta@unito.it (F.F.); roberto.canaparo@unito.it (R.C.); ilaria.andreana@unito.it (I.A.); alessandro.barge@unito.it (A.B.); elena.peira@unito.it (E.P.); silvia.arpicco@unito.it (S.A.); 2 Faculty of Pharmaceutical and Biochemical Sciences, Universidad Mayor de San Andrés, 3239 La Paz, Bolivia; agimenez@megalink.com; 3 National Institute of Metrological Research (INRIM), 10135 Torino, Italy; g.durando@inrim.it

**Keywords:** chlorophyll, liposomes, solid lipid nanoparticles, PLGA, ultrasound, sonodynamic treatment, sonosensitizer, cancer

## Abstract

Sonodynamic therapy is an emerging approach that uses low-intensity ultrasound to activate a sonosensitizer agent triggering its cytotoxicity for selective cancer cell killing. Several molecules have been proposed as sonosensitizer agents, but most of these, as chlorophyll, are strongly hydrophobic with a low selectivity towards cancer tissues. Nanocarriers can help to deliver more efficiently the sonosensitizer agents in the target tumor site, increasing at the same time their sonodynamic effect, since nanosystems act as cavitation nuclei. Herein, we propose the incorporation of unmodified plant-extracted chlorophyll into nanocarriers with different composition and structure (i.e., liposomes, solid lipid nanoparticles and poly(lactic-*co*-glycolic acid) nanoparticles) to obtain aqueous formulations of this natural pigment. The nanocarriers have been deeply characterized and then incubated with human prostatic cancer cells (PC-3) and spheroids (DU-145) to assess the influence of the different formulations on the chlorophyll sonodynamic effect. The highest sonodynamic cytotoxicity was obtained with chlorophyll loaded into poly(lactic-*co*-glycolic acid) nanoparticles, showing promising results for future clinical investigations on sonodynamic therapy.

## 1. Introduction

Sonodynamic therapy (SDT) was introduced by Yumita in 1989 [1]. This recent non-invasive treatment is based on the photodynamic therapy (PDT) already well known in clinical practice, especially in the oncology field [2]. SDT refers to a new anticancer strategy which uses non-thermal ultrasound (US) energy in combination with drugs known as sonosensitizer agents [3,4,5,6]. Normally, low-intensity US frequency are used for this technique (1–3 MHz) to enhance the cavitation effect [7,8]. The sonosensitizer molecule together with US irradiation, under aerobic conditions, create active oxygen species able to destroy the tumor tissues [9,10]. Moreover, the incorporation of sonosensitizers into nanoscaled carriers can increase the therapeutic efficacy of SDT through the enhanced accumulation of drugs in the target tumor site combined with the ability of nanocarriers to function as cavitation nuclei [11,12,13]. Among many sensitizers already investigated, some studies showed that derivatives of chlorophylls/chlorins are potent photosensitizers in PDT [14,15]; on the contrary, natural chlorophyll has not been so deeply exploited yet, and only few papers have proposed it as a sonosensitizer molecule in SDT [16,17,18]. Despite that, the array of photoproperties, biocompatibility and natural abundance of chlorophyll make it a suitable sonosensitizer candidate for SDT application [19], even if the hydrophobicity of the chlorophyll macrocycle is a limit that cuts down the applications in aqueous environment. Therefore, some chlorophyll derivatives have been obtained and associated to colloidal carriers, such as micelles, ethosomes, liposomes, and nanoparticles, but only for PDT applications [20,21,22,23,24,25]. This is the first work that investigates chlorophyll as it is in nature, such as a mixture of different chlorophyll molecules, including chlorophyll *a*, *b* and pheophytin *a*. In particular, herein we used chlorophyll obtained during the isolation of alkaloids from *Galipea longiflora*, a medicinal species also known by the Amazonian ethnic group of Tacana as Evanta. It is traditionally used as an antiparasitic agent in form of cataplasms and decoction. Recently, clinical studies on Evanta alkaloids have showed interesting data in treating cutaneous Leishmaniosis [26,27,28]. In all the purification steps performed to isolate Evanta alkaloids, chlorophyll is considered a non-useful fraction.

Based on these considerations, our aim was to use unmodified plant-extracted chlorophyll and to incorporate it into nanocarriers with different composition (lipidic or polymeric) and different structure (vesicular or matricial) to obtain aqueous chlorophyll-loaded nanosuspensions suitable to act as sonosensitizer agents. The efficacy of these formulations has been tested on human prostatic cancer cells (PC-3) and spheroids (DU-145) and the reactive oxygen species (ROS) production has been determined.

## 2. Materials and Methods

### 2.1. Materials and Instruments

All the phospholipids were provided by Avanti Polar Lipids and distributed by Sigma-Aldrich (Milan, Italy). Miglyol 812N was a gift from Sasol (Witten, Germany). Poly(lactide-*co*-glycolide) (PLGA) 75:25 (Resomer RG 752 H), trilaurin, ethyl acetate, benzyl alcohol, sodium taurodeoxycholate, Pluronic F68, Sepharose CL-4B, cholesterol and all other reagents were obtained from Sigma-Aldrich. Cremophor RH 60 (PEG-60 hydrogenated castor oil) was purchased from BASF (Ludwigshafen, Germany). All the solvents used were of analytical grade and purchased from Carlo Erba Reagenti (Milan, Italy). Solvent evaporation was carried out using a rotating evaporator (Heidolph Laborota 400, Heidolph Instruments, Schwabach, Germany) equipped with a vacuum pump (Diaphragm Vacuum Pump DC-4). TLC analyses were performed on silica gel aluminium plates (Macherey-Nagel, thick 25 µm, F254). HPLC analyses were carried out on a Waters instrument made up of 1525EF binary pump, W717 plus auto-sampler and 2996 PDA detector (Waters, Milan, Italy).

### 2.2. Chlorophyll Extraction

Thanks to the help of the Tacana community, Evanta leaves were picked up by Gimenez’s group (Instituto de Investigaciones Fármaco-Bioquímicas (IIFB), La Paz, Bolivia) in Sud Yungas area in La Paz district [28,29]. The taxonomical identification was made by comparing the plant with samples coming from the Herbario Nacional de Bolivia. Evanta leaves were air-dried for several days at room temperature, in the dark and protected from humidity. Ethanol (25 L) was used as solvent to extract milled material (5 kg). After one week, the filtrate was evaporated obtaining a residue (6–10 g), which was dissolved in ethanol (100 mL). The solution was extracted with petroleum ether (2 × 100 mL) and the collected organic phases were dried over sodium sulphate and evaporated to give 3 g of crude product, which was re-dissolved in petroleum ether (80 mL) and washed with methanol (2 × 80 mL). Sodium sulphate was used to dry petroleum ether phase and then the solvent was removed under reduced pressure obtaining a green sticky solid (2.5 g). First, purification was performed on a Sephadex LH 20 column (26 × 560 mm) eluting with chloroform/methanol 1:1, v/v. Fractions were collected into 5 clusters by UV-Vis analysis. Each cluster was analyzed by HPLC on a Waters XTerra phenyl column (4.6 × 150.5 µm), using water (0.1% trifluoroacetic acid) and methanol (0.1% trifluoroacetic acid) as eluents. Gradient profile was set as follows: (min, %B) 0, 65; 15.0, 65; 27.4, 100; 42.4, 100. Wavelength range observed by PDA detector was between 210 and 700 nm. Further HPLC analysis (eluted with methanol 100%) showed a very similar composition (three main peaks with the same shape were present) only in terms of chlorophyll content (at 405 nm, maximum absorbance wavelength typical of chlorophylls) for all analyzed fractions (data not shown). A second purification by Sephadex LH 20 column (1.5 × 26 cm; mobile phases 20%, 50%, 80% ethanol in water) was carried out. Two final fractions were isolated: one retained/held on Sephadex LH 20 and one eluted with ethanol 100% (washing phase). The content of chlorophylls was the same (in terms of peaks profile) in both fractions. The chlorophyll mixture (A and B) (Chlor) was stored at −20 °C in tert-butilmethylether. For the analysis, the sample was dissolved in acetonitrile/methanol/water 70:20:5, v/v. Reverse phase chromatography column C30 (YMC) was used. DAD at 680, 480 e 420 nm and ESI-Ion trap MS2 in Multi Reaction Monitoring modality was the set up for the detection of the desired molecules.

### 2.3. Preparation of Nanocarriers

#### 2.3.1. Liposomes

Chlor-containing liposomes (Chlor-Lipo) were obtained by the method of thin lipid film hydration and extrusion. Practically, a chloroform solution of the lipid components 1,2-distearoyl-*sn*-glycero-3-phosphocholine (DSPC), cholesterol (CHOL) and 1,2-distearoyl-*sn*-glycero-3-phosphoethanolamine-*N*-[amino(polyethylene glycol)-2000] (DSPE-PEG, ammonium salt) (78:16:6 molar ratios) and containing 12% Chlor (mol Chlor/mol lipid) was dried by a rotating evaporator. The obtained lipid film was vacuum dried overnight, then hydrated with a 20-mM 4-(2-hydroxyethyl)piperazine-1-ethanesulforic acid (HEPES) buffer pH 7.4; the suspension was vortexed for 10 min and then bath sonicated. The formulations were then passed 5 times under nitrogen through a 400 and then a 200 nm polycarbonate membrane (Costar, Corning Incorporated, Corning, NY, USA) in an extruder (Extruder, Lipex, Vancouver, BC, Canada) at a set temperature of 5 °C above the phase transition temperature of the lipid mixture. Unentrapped Chlor was separated through chromatography on Sepharose CL-4B columns, eluting with HEPES buffer. Liposomes were stored in the dark at 4 °C.

#### 2.3.2. Solid Lipid Nanoparticles (SLN)

Chlor-containing SLN (Chlor-SLN) were prepared by the “cold dilution of microemulsion” method. This method involves the preparation of an oil/water microemulsion (µE) and the subsequent dilution with a 2% w/w polymeric aqueous solution to precipitate SLN [30]. Two hundred microliters (200 µL) of trilaurin solution in ethyl acetate (300 mg/mL) was chosen as an oily phase since this lipid is highly soluble in this partially water miscible solvent. Ethyl acetate and water were mutually pre-saturated (named EA_s_ and water_s_, respectively) and then used in µE formulation. Twelve percent (12%) w/w Epikuron 200 (phosphatidyl choline 92%) was chosen as a surfactant together with Cremophor RH 60 at 3:1 w/w constant ratio. Then, 2.5% w/w sodium taurodeoxycholate was tested as co-surfactant and 4% w/w benzyl alcohol as a co-solvent to pre-solubilize Chlor. Chlor was added to the µE up to 0.6% w/w. µE (1.2 mL) was then diluted using an aqueous solution of Pluronic F68 2% w/w (5 mL); the organic solvent was removed from the disperse phase and was extracted by dissolution into the continuous phase, determining the 1% w/w trilaurin-SLN precipitation. SLN were purified from unentrapped compound through chromatography on Sepharose CL-4B columns, eluting with a hypertonic phosphate buffer saline (PBS) buffer (NaCl/KCl molar ratio 2:1). Fractions containing purified SLN were collected and concentrated for the determination of the entrapped Chlor amount. Then, the SLN dispersion was stored in the dark at 4 °C.

#### 2.3.3. PLGA Nanoparticles

For the preparation of Chlor-containing PLGA 75:25 nanospheres (Chlor-PLGA NSs) and nanocapsules (Chlor-PLGA NCs) the nanoprecipitation technique was employed [31]. Practically, for each preparation of Chlor-containing NSs, 24 mg of PLGA 75:25 were solubilized in acetone and an aliquot of a stock solution of Chlor in ethanol (8 mg/mL) was added to obtain a total volume of 2 mL. This organic solution was then added to 4 mL of MilliQ^®^ water under stirring. Particles formed spontaneously. After solvent evaporation by a rotating evaporator, NSs in water were obtained. For Chlor-containing NCs preparation, the same method was used, except that we added 4 µL of Miglyol 812N to the polymer organic solution to form the inner oily cavity. To separate the nanocarriers from unentrapped Chlor, they were eluted through Sepharose CL-4B columns with PBS buffer. The particles were then stored in the dark at 4 °C.

### 2.4. Physico-Chemical Characterization of Nanocarriers

The average diameter and the polydispersity index (PDI) of liposomes, SLN and PLGA NSs and NCs were determined by quasi-elastic light scattering (QELS) at 25 °C with a nanosizer (Nanosizer Nano Z, Malvern Inst., Malvern, UK). The selected angle was 90° and the measurements were made after dilution of the particle suspensions in MilliQ^®^ water. Each value reported is the average of three measurements.

The particle surface charge of all formulations was investigated by zeta potential measurements at 25 °C by diluting the suspensions in 10-mM KCl using the Smoluchowski equation and the Nanosizer Nano Z. Three independent measures were performed.

The amount of Chlor incorporated in nanocarriers was determined by means of UV-Vis measurements (Beckman Coulter DU730, Beckman Coulter, Milan, Italy) at 400 nm and at room temperature (calibration curve obtained with dilutions of a Chlor stock solution in methanol in the range 10–200 µg/mL). The results are the average of three measurements and they were expressed as encapsulation efficiency (EE), calculated as the ratio between the amount of entrapped Chlor and the initial amount used in the preparation of nanocarriers × 100. Moreover, drug loading (DL) was calculated as the ratio between the amount of entrapped Chlor and the total nanocarrier weight × 100. In particular, for liposomes, to 800 µL of suspension 200 µL of methanol were added and the sample was centrifuged (6000 rpm, 15 min) and measured spectrophotometrically. Moreover, phospholipid phosphorous was evaluated in each preparation performing the phosphate assay after destruction with perchloric acid [32]. For SLN, the suspension was diluted 1:40 with a mixture of dichloromethane/methanol (1:1, v/v) and after centrifugation (14,000 rpm, 15 min), the supernatant was measured spectrophotometrically. For PLGA nanocarriers, 2 mL of previously prepared PLGA NSs or NCs were lyophilized. Then, 200 µL of dichloromethane were added to dissolve the sample and then 2 mL of methanol to precipitate PLGA. After centrifugation, 500 µL of the supernatant were diluted 1:2 with methanol and measured spectrophotometrically.

The physical colloidal stability of all the Chlor-containing suspensions in the storage conditions was evaluated by measuring the mean size, the zeta potential and the drug content of the particles over a storage period of 28 days at 4 °C.

Chlor-encapsulating carriers were tested by differential scanning calorimetry (DSC) using a differential scanning calorimeter DSC 7 (Perkin-Elmer, Waltham, MA, USA) equipped with an instrument controller Tac 7/DX (Perkin-Elmer). Indium was used to calibrate the instrument daily (ΔH = 28.4 J/g, m.p. 156.6 °C) for the determination of melting point and heat of fusion. A heating rate of 10 °C/min was employed throughout the analyses to assess the melting point and the heat of fusion of particles. The temperature range in which thermograms were recorded was 28–170 °C. Analyses were performed under a nitrogen purge (40 mL/min); standard 40 µL aluminum sample pans (Perkin-Elmer) were used; as reference, an empty pan was used. The main transition temperature (T_m_) was determined as the onset temperature of the highest peak. Indium (T_m_ = 156.83 °C) and *n*-decane (T_m_ = −29.6 °C) were used to obtain the calibration. Triple runs were carried out on each sample. For liposomes, about 10 mg of suspension samples were introduced into an aluminum pan and analyzed. SLN and PLGA suspensions were freeze-dried using a Modulyo Freeze Dryer (Edwards Alto Vuoto, Cinisello Balsamo, Italy). Freeze-dried nanoparticles were weighted and placed in the aluminum pan for analysis.

### 2.5. Drug Release

The drug release was determined by incubating the nanocarrier suspensions in HEPES (Chlor-Lipo) or PBS (Chlor-SLN and Chlor-PLGA NSs) buffer pH 7.4 at 37 °C. At different time intervals aliquots of the formulations were submitted to purification, re-analyzed for Chlor content as described above and compared with initial values. Sink conditions were maintained by replacing the removed aliquots with the same quantity of fresh buffer.

### 2.6. Cell Culture

The human prostatic carcinoma cell lines PC-3 and DU-145 (ICLC, Interlab Cell Line Collection, Genoa, Italy) and the human dermal fibroblast cell line HDF 106-05 (ECACC, Salisbury, UK) were grown in Dulbecco’s modified Eagle medium (DMEM) added with 2-mM L-glutamine, 100 UI/mL penicillin, 100 µg/mL streptomycin and 10% v/v heat-inactivated fetal calf serum, and incubated in 5% CO_2_ air at 37 °C. When cells were grown to confluence, they were detached using a 0.05% trypsin-0.02% EDTA solution and seeded at the appropriate cell density in culture medium for the different cell culture experiments.

### 2.7. Cytotoxicity

The WST-1 cell growth assay was carried out to determine the cytotoxic effect on PC-3 cells of Chlor, to select the concentration suitable for the in vitro sonodynamic treatment. Briefly, in 96-well culture plates (TPP, Trasadingen, Switzerland) 1.5 × 10^3^ cells per well were seeded in 100 μL of culture medium in 8 replicates; after 24 h, the medium was removed and the cells incubated with experimental media of differing Chlor concentrations (5, 10, 50, 100 and 500 µM) obtained by diluting in DMEM a 50 mM Chlor DMSO solution. The WST-1 reagent (10 μL/100 µL) was added at 24, 48, and 72 h and the plates were incubated at 37 °C in 5% CO_2_ in air for 90 min. A microplate reader (Asys UV340, Biochrom, Cambridge, UK) was used to detect well absorbance at 450 and 620 nm (reference wavelength).

### 2.8. In Vitro Sonodynamic Treatment

1 × 10^5^ PC-3 cells were sub-cultured into Petri dishes (25 mm diameter) and were allowed to attach to the surface for 24 h prior to treatment. Free Chlor or the Chlor-containing formulations were then added to the cell culture medium to the appropriate concentration (i.e., 5 µM Chlor) and incubated for the proper time according to the Chlor cell uptake analysis (i.e., 6 h for Chlor-Lipo and Chlor-SLN or 24 h for Chlor and Chlor-PLGA NSs), before ultrasound (US) exposure. The US field was generated by a plane wave transducer (25 mm diameter) at 1.5 MHz, connected to a power amplifier (Type AR 100A250A, Amplifier Research, Souderton, PA, USA) and a function generator (Type 33250, Agilent, Santa Clara, CA, USA). A mechanical adaptor was built to connect the Petri dish containing the cells and filled with ultrapure water to create highly reproducible measurement conditions, at a fixed Petri dish distance from the transducer (20 mm). Specifically, US exposure was performed at 1.5 MHz for 5 min at 1.5 W/cm^2^ under a dim light. After US exposure, PC-3 cells were removed with a 0.05% trypsin-0.02% EDTA solution and in 96-well culture plates were seeded 1.5 × 10^3^ cells per well in 100 µL of culture medium in replicates (*n* = 8). The WST-1 reagent (10 µL/100 µL) was added at 24 and 48 h, and plates were incubated at 37 °C in 5% CO_2_ for 90 min. The microplate reader Asys UV340 was used to detect well absorbance at 450 and 620 nm (reference wavelength). Results were expressed as the percentage of the absorbance of treated versus untreated cells (100%). Moreover, the effect of Chlor-PLGA NSs under US treatment was also explored on the non-cancerous cell line HDF 106-05, under the same US treatment conditions previously described. Cell growth was then evaluated at 24 and 48 h by WST-1 reagent.

### 2.9. Flow Cytometric Analyses

A C6 flow cytometer (Accuri Cytometers, Milan, Italy) was used for the cellular uptake analysis of free Chlor and Chlor-containing formulations. Briefly, PC-3 cells were seeded in 6-well culture plates (TPP) at 5 × 10^4^ per well and incubated with each Chlor formulation (5 µM) for 1, 3, 6, 12 and 24 h. Moreover, the cellular uptake of Chlor and Chlor-PLGA NSs was also performed in the human prostate cancer DU-145 cells at the same incubation time. At the end of each incubation period, cells were re-suspended in 300 µL PBS after detachment by a 0.05% trypsin-0.02% EDTA solution. For the cytofluorimetric analysis of the intracellular Chlor, 10,000 events were considered, using 640-nm excitation. The integrated mean fluorescence intensity (iMFI) was used to express the intracellular fluorescence as the product of the frequency of the mean fluorescence intensity of the cells and the frequency of cells positive to Chlor fluorescence. Data are expressed as iMFI ratio, i.e., the ratio between the iMFI of treated and untreated cells. The proper Chlor incubation time for the US exposure was then chosen according to the iMFI ratio.

Reactive oxygen species (ROS) production was evaluated 1, 15, 30, 60 and 90 min after US treatment of Chlor pre-incubated PC-3 cells. Cells were incubated with 10 µM of 2,7-dichlorofluorescein diacetate (DCFH-DA) as intracellular probe for oxidative stress, 30 min before the flow cytometric analysis. DCFH-DA is a nonfluorescent molecule able to promptly cross the cell membrane and to be hydrolyzed to the non-fluorescent DCFH by intracellular esterases. DCFH is rapidly oxidized to highly fluorescent dichlorofluorescein (DCF) upon oxidation by ROS. After DCFH-DA incubation, ROS generation was carried out by the C6 flow cytometer, evaluating 10,000 events at a 488-nm excitation. ROS production was expressed as iMFI ratio, where the iMFI is the product of the mean fluorescence intensity of the cells and the frequency of ROS-producing cells.

PC-3 cell death was evaluated using the allophycocyanin (APC)-Annexin V and propidium iodide (PI) Apoptosis Detection Kit (Life Technologies, Milan, Italy) 24 h after the treatment. Briefly, 1.0 × 10^5^ PC-3 cells were detached by trypsin, washed with 1× Annexin-binding buffer and stained with APC-Annexin V and PI. Samples were analyzed by using the C6 flow cytometer, considering 10,000 events and any cell debris were excluded from the analyses. All analyses were carried out using FCS Express software version 4 (BD Bioscience, Italy).

### 2.10. ROS Scavenging Assay

*N*-acetyl-cysteine (NAC, Sigma-Aldrich, Milan, Italy) was used to evaluate ROS role to provoke cytotoxicity under US exposure in Chlor-incubated PC-3 cells. Specifically, PC-3 were incubated for 6 h with Chlor-Lipo or Chlor-SLN and for 24 h with Chlor and Chlor-PLGA NSs at the same concentration (5 µM Chlor) and 5 mM NAC was added for the last 3 h of Chlor incubation. Cells were then detached from the flask, washed with PBS and treated with US, using the settings and parameters previously mentioned. WST-1 was then used to determine cell growth after 24 and 48 h.

### 2.11. Fluorescence Microscopy

The uptake of the various Chlor formulations by PC-3 cells was determined by using fluorescence microscopy in order to obtain a qualitative intracellular localization of compounds. Briefly, 2 × 10^5^ PC-3 were seeded for 24 h on glass coverslips in small plates and then underwent to Chlor-Lipo and Chlor-SLN incubation for 6 h or to Chlor and Chlor-PLGA NSs incubation for 24 h (5 µM Chlor concentration). At the end of the incubation time, slides were fixed for 15 min with 4% paraformaldehyde and 4′,6-diamidine-2′-phenylindole dihydrochloride (DAPI) was added for 10 min to stain cell nuclei. Images were acquired using a Leica DMI4000B fluorescence microscope (Leica Microsystems, Milan, Italy).

### 2.12. DU-145 Spheroid Generation

DU-145 spheroids were grown in 96-well plates (U shape, Perkin Elmer). Cells were seeded (6 × 10^3^, 200 μL) in selected wells previously coated with agarose (1.5%, Sigma-Aldrich). Plates were then put inside the incubator for a total of 6 days in order to let cells to organize in 3D structure. Every 3 days, the cellular medium was changed by replacing 100 μL of old media with 100 μL of fresh media. Six days after the seeding, spheroids were selected and divided into the following groups: control (i.e., untreated, molecule-free medium), Chlor only (10 μM) and Chlor-PLGA NSs (10 μM Chlor). At the end of the incubation, where required, individual spheroids were washed with PBS and then exposed to US. At the end of each treatment, spheroids were incubated in a humidified 5% CO_2_ atmosphere at 37 °C. Twenty-four hours after treatment, cellular damage on the corona was investigated by staining spheroids with PI. Briefly, spheroids underwent to 4 PBS washes to remove medium and were then incubated in the dark at room temperature with PI (100 μg/mL) in PBS for 20 min. At the end of the incubation, spheroids were washed 3 times with PBS to remove PI excess and then images were captured using a Leica DM6000 fluorescent microscope. Bright and fluorescence fields (540 nm band pass excitation and 590 nm long pass emission filters) were acquired. PI fluorescence quantification was then determined by using Image J software and it was expressed as a % of PI fluorescence intensity/μm^2^, i.e., the fluorescence of PI was normalized and adjusted according to the spheroid area.

### 2.13. Statistical Analysis

In vitro data are shown as average values of three independent experiments ± standard deviation. Statistical analyses were performed using Graph-Pad Prism 5.0 software (La Jolla, CA, USA); one- and two-way analyses of variance and Bonferroni’s test were used to calculate the threshold of significance. Statistical significance was set at *p* < 0.05.

## 3. Results

### 3.1. Chlorophyll Extraction

**Figure 1:**
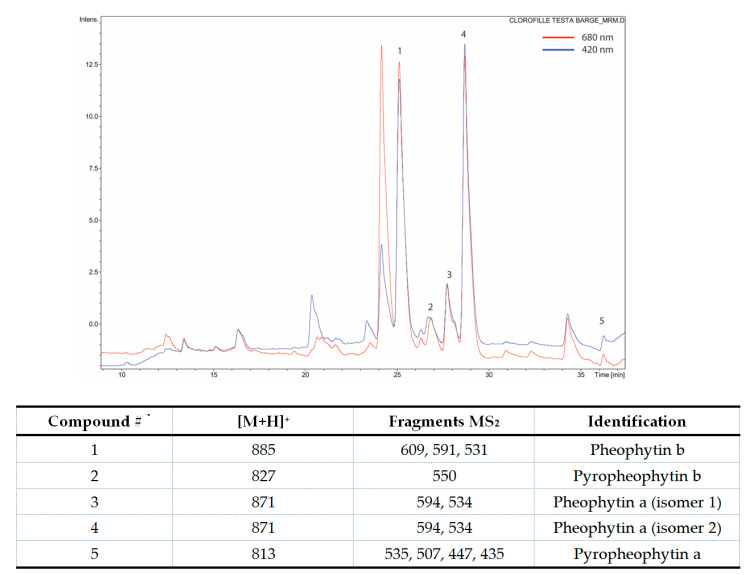
HPLC-MS chromatogram with [M + H]^+^ mass fragments identification. Chlor for the analysis was dissolved in acetonitrile/methanol/water 70:20:5, v/v.

Chlor was obtained from leaves of *Galipea longiflora* by extraction and purification. According to the detection analysis of the obtained molecules, at 420 nm the predominant mass fragmentations were identified as pheophytin a, b (Figure 1).

### 3.2. Preparation and Characterization of Nanocarriers

Chlor-loaded nanocarriers of different composition (liposomes, SLN and PLGA nanoparticles) were prepared to compare the Chlor encapsulation efficiency and the in vitro efficacy on cancer cells. The physico-chemical characteristics of the nanocarriers are summarized in Table 1.

Different liposomal formulations were tested and liposomes composed of DSPC:CHOL:DSPE-PEG (78:16:6) showed the best characteristics in term of Chlor EE (98%), corresponding to a DL of 1.8%. We observed that the presence of PEG stabilized the formulation. Liposomes displayed a dimensional range around 165 nm with a low PDI (<0.13) and a negative zeta potential value around −6 mV (Table 1).

Chlor-SLN mean diameter was about 250 nm, higher than that of liposomes, with a low PDI, suggesting the presence of a monodispersed size population. Chlor-SLN showed a zeta potential of about −20 mV and a high EE (98%) (DL 9.1%), indicating that the new SLN preparation method from µE avoids significant Chlor losses during the formulation (Table 1).

Concerning Chlor-loaded PLGA 75:25 nanoparticles, the mean diameter of NCs was higher (about 170 nm) than that of NSs (140 nm), as expected, due to the presence of the inner oily cavity formed by Mygliol 812N. Nevertheless, both formulations showed a unimodal size distribution (PDI < 0.1). Due to the negative charges of PLGA 75:25 copolymer, Chlor-containing NSs and NCs both displayed highly negative zeta potential values. While, for Chlor-PLGA NSs, the EE was similar to that of liposomes and SLN, for NCs it was below 90% (Table 1), corresponding to DL values of 4.6% and 3.6%, respectively.

The physical stability of all the Chlor-containing suspensions in the storage conditions was evaluated by measuring the mean size and the zeta potential of the particles over a storage of 28 days: during this period no precipitation, aggregation or appreciable formulation size or zeta potential modifications occurred (less than 10% for all the samples), excepted for Chlor-PLGA NCs, whose mean diameter began to increase after 3 weeks (>250 nm). Considering the lower EE and stability of PLGA NCs if compared to NSs, we decided to carry out further assays only with PLGA NSs. The drug leakage during the storage was determined by removing aliquots of liposomes, SLN or PLGA NSs at various time points and re-determining after purification the drug content; the formulations maintained their initial drug content for at least 90% of the initial value.

In order to evaluate the interactions between Chlor and the nanocarrier components, DSC analysis was performed for all Chlor-containing formulations. Concerning liposomes phospholipids, the main transition of pure DSPC was at T_onset_ = 54.2 °C. With DSPE-PEG, the transition temperature did not significantly change (T_onset_ = 53.9 °C), but the peak was slightly enlarged. The incorporation of CHOL altered the calorimetric profile: the main transition was shifted to a lower temperature (T_onset_ = 51.6 °C), indicating the interaction of CHOL with the liposome bilayer. When Chlor was encapsulated, the main transition was found at a lower temperature (T_onset_ = 47.8 °C) and the melting temperature peaks were broadened, indicating that Chlor through hydrophobic interactions perturbs the phase transition profile (Table 2). To better understand the interaction of Chlor with the main phospholipid in the liposomes (DSPC), two formulations with different amounts of Chlor (6% and 12% mol) were prepared. In the formulation containing 6% of Chlor the main transition occurred at a higher temperature if compared to the one containing 12% Chlor (52.7 and 49.8 °C, respectively), revealing a Chlor concentration-dependent effect on the main transition temperature of DSPC. A similar trend was observed in thermal phase behavior studies on liposomes containing temoporfirin, a potent, clinically approved, second-generation photosensitizer [33].

For SLN, DSC analysis was aimed to verify the solid state after µE dilution and to evaluate the degree of crystallinity of lipids, investigating the presence of polymorphs, since these conditions influence the drug incorporation and release profiles [34]. Trilaurin, a triglyceride obtained by esterification of glycerol with lauric acid, shows a sharp endotherm peak at T_onset_ = 48 °C, corresponding to the melting of the stable trilaurin β-form [35]. DSC thermograms of Chlor-SLN showed a broaden endotherm peak with T_onset_ = 42.6 °C, probably related to the unstable β′-form melting and no supercooled melt was revealed (Table 2). Alternatively, according to Siekmann and Westesen [36], the shift of the melting point to a lower temperature for SLN might be due to their colloidal nature, in particular to their high surface-to-volume ratio and not to recrystallization of the lipids in a metastable polymorph.

Unloaded PLGA 75:25 NSs showed a sharp main endothermic event with T_onset_ = 52.3 °C and glass transition temperature 54.2 °C. Incorporation of Chlor into PLGA NSs slightly decreased the melting temperature (Table 2).

The Chlor release profile from the different formulations was evaluated in HEPES (Chlor-Lipo) or PBS (Chlor-SLN and Chlor-PLGA NSs) buffer at 37 °C. During 24 h, the release was faster for liposomes and SLN (40% and 35% of the initial Chlor content, respectively) than for PLGA NSs (18%).

### 3.3. PC-3 Cell Uptake of Chlor-Containing Formulations

**Figure 2:**
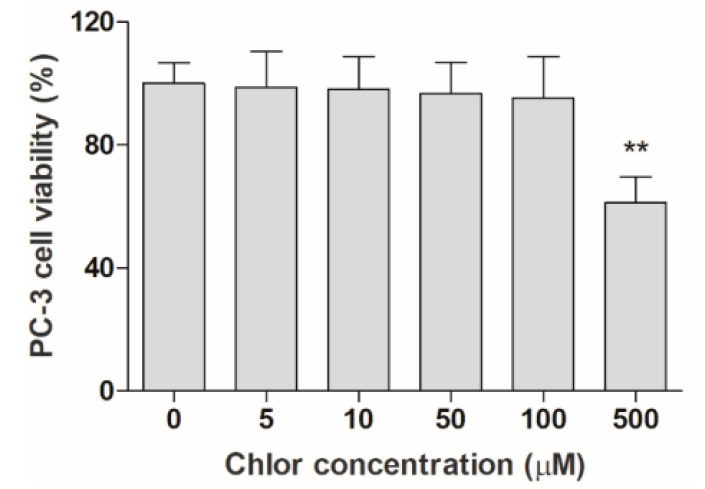
Effect of Chlor on PC-3 cells. Cell viability, after exposure to increasing concentrations of Chlor (5, 10, 50, 100 and 500 μM), was evaluated at 24 h by WST-1 assay. Statistical significance vs. untreated cells: ** *p* < 0.01.

To investigate the efficacy of the different Chlor formulations, namely Chlor-Lipo, Chlor-SLN and Chlor-PLGA NSs, as sonosensitizers, we first investigated the Chlor cytotoxicity to select a proper non-cytotoxic concentration. As shown in Figure 2, Chlor started to significantly affect PC-3 cell growth at 500 μM. Thus, we decided to investigate the Chlor efficacy as sonosensitizer at a 100-times lower concentration than the cytotoxic concentration observed, i.e., 5 μM, being the sonosensitizer non-cytotoxicity itself one of the pivotal features of the sonodynamic treatment.

**Figure 3:**
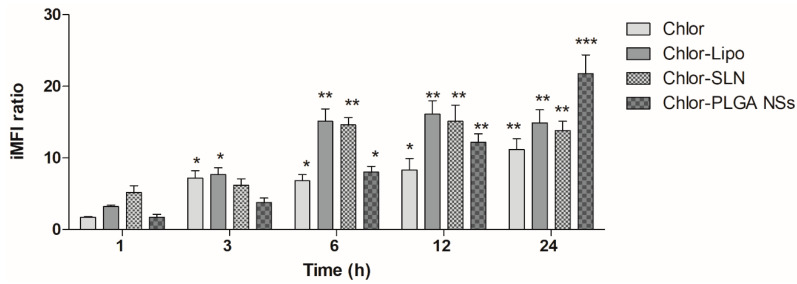
Chlor uptake by PC-3 cells. PC-3 cells were incubated with Chlor formulations at the same Chlor concentration (5 μM) for 1, 3, 6, 12 and 24 h. Fluorescent signal was detected using a flow cytometer at 488 nm excitation to measure the intracellular Chlor and expressed as the integrated mean fluorescence intensity (iMFI) ratio calculated as reported in Materials and Methods. Statistical significance vs. 1 h incubation: * *p* < 0.05; ** *p* < 0.01; *** *p* < 0.001.

To investigate the influence of the different Chlor formulations on the PC-3 cell uptake, we performed a flow cytometric study at the same non-cytotoxic Chlor concentration, i.e., 5 μM, for all the formulations. A time-dependent increase of the iMFI ratio, corresponding to the Chlor internalization, was observed after the incubation of all the Chlor formulations (Figure 3). Noteworthy is the fact that the iMFI was significantly enhanced for the encapsulated Chlor compared to the free Chlor after 6 and 12 h incubation for liposomes and SLN, and after 12 and 24 h incubation for PLGA NSs. The higher statistically significant level of Chlor cell uptake was reached after 6 h incubation for Chlor-Lipo (iMFI ratio 15.1 ± 1.7, *p* < 0.01) and Chlor-SLN (iMFI ratio 14.6 ± 1.0, *p* < 0.01) and after 24 h incubation for Chlor (iMFI ratio 11.2 ± 1.5, *p* < 0.01) and Chlor-PLGA NSs (iMFI ratio 21.8 ± 2.6, *p* < 0.001). Therefore, these incubation times were selected for the sonodynamic treatment with the different Chlor formulations.

**Figure 4:**
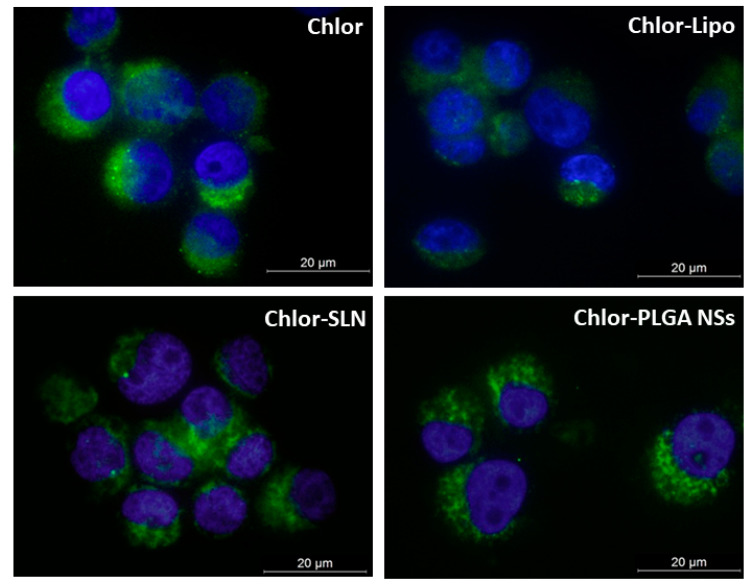
Representative fluorescence images of PC-3 cells incubated with the various Chlor formulations. Cells were incubated for 6 h with Chlor-Lipo or Chlor-SLN and for 24 h with Chlor or Chlor-PLGA NSs at the same Chlor concentration (5 μM); DAPI (blue) was used a nuclear counterstain. Magnification: 63×. Scale bars: 20 μm.

Moreover, to discriminate differences in the Chlor intracellular localization due to the different formulations used, we observed PC-3 cells by fluorescence microscopy. Figure 4 shows principally a cytoplasmic distribution of Chlor for all the formulations tested at the selected times of incubation (6 h for Chlor-Lipo and Chlor-SLN, and 24 h for Chlor and Chlor-PLGA NSs).

### 3.4. Effect of the Sonodynamic Treatment with the Chlor Formulations on PC-3 Cell Growth, ROS Production and Cell Death

**Figure 5:**
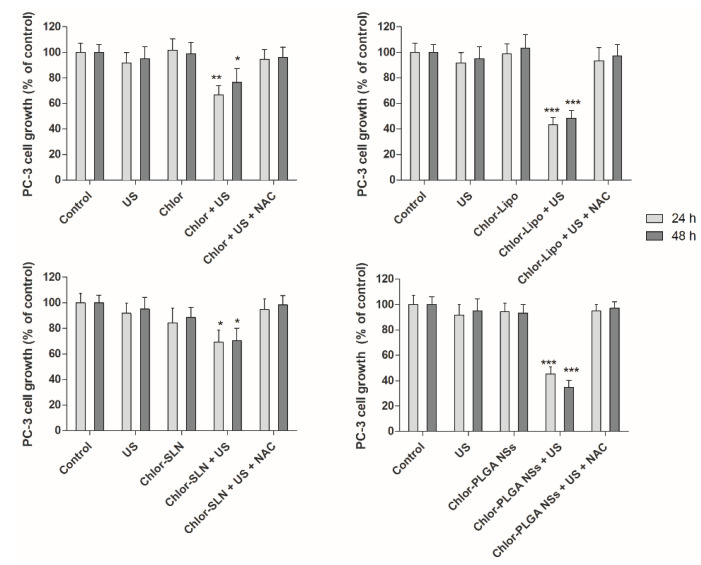
Effect of sonodynamic treatment on PC-3 cell growth. Cells were incubated for 6 h with Chlor-Lipo or Chlor-SLN and for 24 h with Chlor or Chlor-PLGA NSs at the same concentration (5 μM) and then exposed to US at 1.5 MHz for 5 min at 1.5 W/cm^2^. In the graphs is also shown the effect of sonodynamic treatment in presence of the ROS scavenger, *N*-acetyl-cysteine (NAC). Before exposing PC-3 cells to US, 5.0 mM NAC was added for the last 3 h incubation of the various Chlor formulations, as reported above. Cells were then exposed to US at 1.5 MHz for 5 min at 1.5 W/cm^2^. Cell growth was evaluated after 24 and 48 h by WST-1 assay. Statistically significant difference vs. untreated cells: * *p* < 0.05, ** *p* < 0.01, *** *p* < 0.001.

The sonodynamic effect on PC-3 cell growth was evaluated 24 and 48 h after US exposure of the prostatic cancer cells pre-incubated for 6 h with Chlor-Lipo or Chlor-SLN and 24 h with Chlor or Chlor-PLGA NSs at the same Chlor concentration (5 μM). As shown in Figure 5, only when the cells were pre-incubated with one of the Chlor formulations the US were able to significantly decrease PC-3 cell growth. Moreover, the decrease in cell growth was time-independent for all the Chlor formulations. Noteworthy was the fact that the higher decrease in cell growth was obtained by the sonodynamic treatment with Chlor-Lipo and Chlor-PLGA NSs, reaching after 48 h a 48.4 ± 6.2% of cell growth with Chlor-Lipo (*p* < 0.001) and a 34.8 ± 5.4% of cell growth with Chlor-PLGA NSs (*p* < 0.001), compared to untreated cell growth.

**Figure 6:**
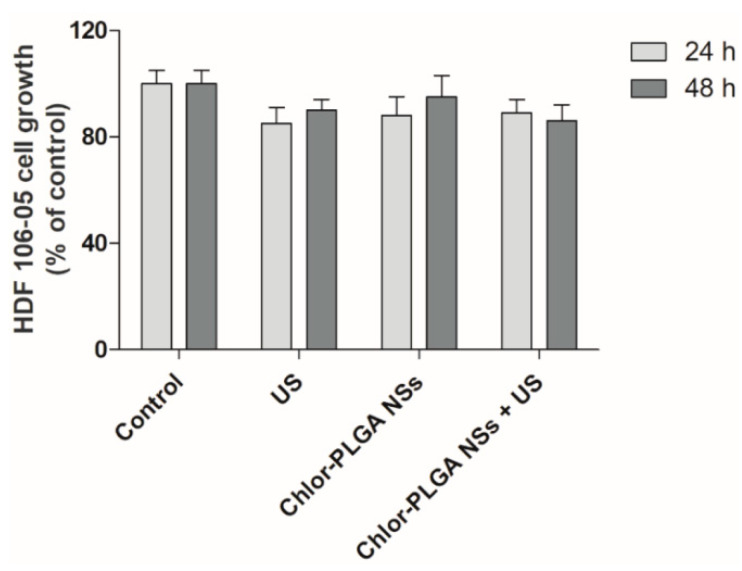
Effect of sonodynamic treatment on HDF 106-05 cell growth. Cells were incubated for 24 h with Chlor-PLGA NSs at the same concentration (5 μM) and then exposed to US at 1.5 MHz for 5 min at 1.5 W/cm^2^. Cell growth was evaluated after 24 and 48 h by WST-1 assay.

Interestingly, cells exposed to each of the Chlor formulations alone or to US alone did not affect PC-3 cell growth when compared with untreated cells, i.e., control cells (Figure 5). Moreover, we did not observe any difference in term of cell growth over time. This could be due to an effect of sonodynamic treatment on cell growth that is not time related. The different activities observed with the Chlor formulations after US exposure might be ascribed to the Chlor cell uptake, as it is lower for Chlor alone with respect to the other formulations (Figure 3), but also to the physico-chemical properties of the different Chlor formulations, as the Chlor cell internalization at the concentration tested of Chlor-Lipo, Chlor-SLN and Chlor-PLGA NSs was quite the same. These can be related to the interaction of the intracellular energy locally generated by US with the nanocarrier structure. Indeed, is well known that the presence of nanocarriers can elicit a cavitation threshold decrease improving the efficacy of the sonodynamic treatment [37,38]. Moreover, we monitored the effect of Chlor-PLGA NSs under US stimulation on the non-cancerous cell line HDF 106-05 over time. We did not observe any significant differences compared to untreated cells (Figure 6), highlighting a selective responsiveness to sonodynamic treatment of cancer cells compared to healthy cells.

Since the mechanism underlying the sensitizer cytotoxicity under US exposure is thought to be ROS generation, we performed the sonodynamic treatments with the different Chlor formulations also in the presence of the ROS-scavenger NAC. Interestingly, when cells were pre-incubated with NAC and then exposed to US, the cytotoxicity of the sonodynamic treatment was suppressed with each one of the Chlor formulations tested (Figure 5, the far-right bars of each graph).

**Figure 7:**
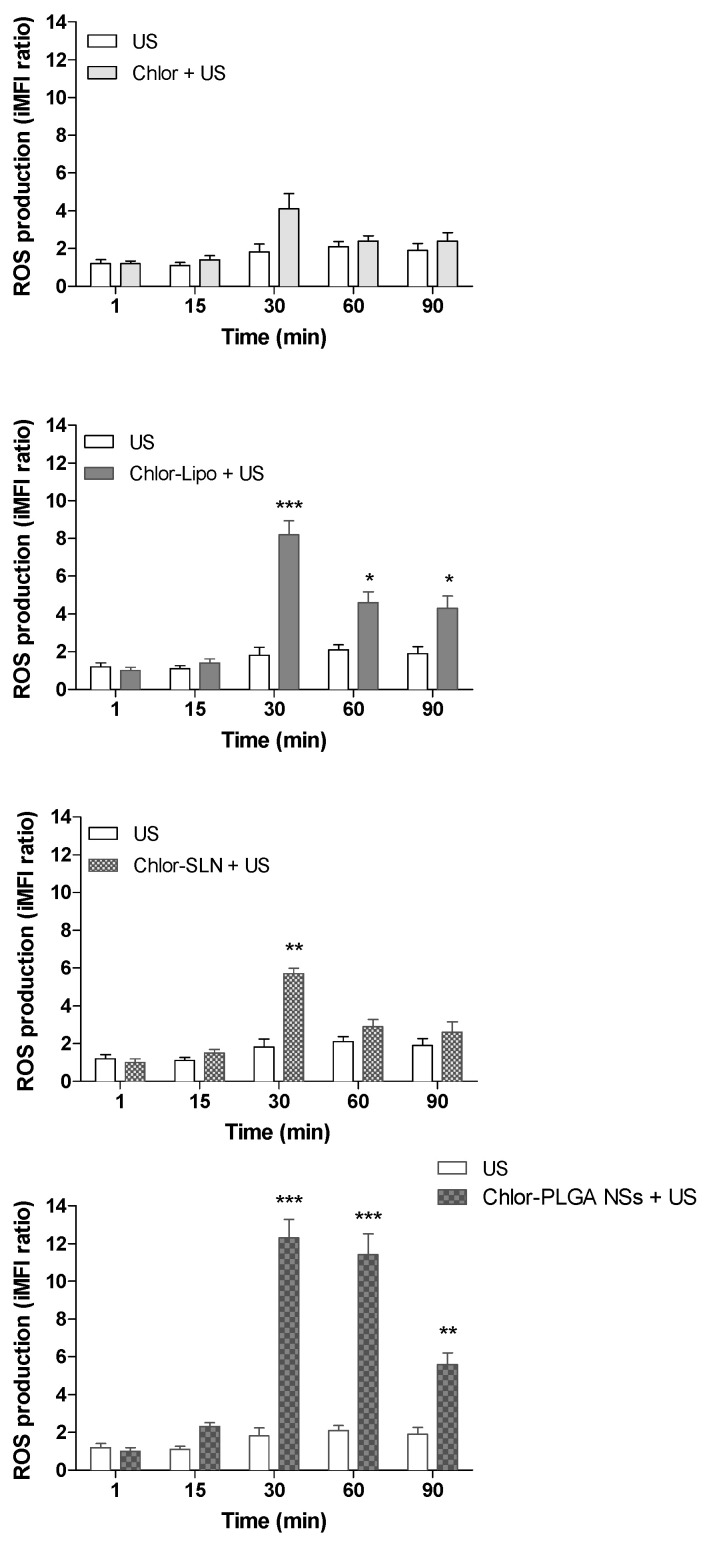
PC-3 ROS production after sonodynamic treatment as a function of time. PC-3 cells were exposed to US (1.5 MHz for 5 min at 1.5 W/cm^2^) or pre-incubated with the various Chlor formulations at the same concentration (5 μM) and the proper time (6 h for Chlor-Lipo or Chlor-SLN and 24 h for Chlor or Chlor-PLGA NSs) and then exposed to US (1.5 MHz for 5 min at 1.5 W/cm^2^). ROS levels were determined according to the DCFH-DA assay by flow cytometry and expressed as the integrated mean fluorescence ratio (iMFI), as described in Materials and Methods. * *p* < 0.05, ** *p* < 0.01, *** *p* < 0.001.

Furthermore, in order to confirm the ROS involvement in the cytotoxicity exerted by the sonodynamic treatment with the different Chlor formulations, we assessed the ROS generation. When PC-3 cells were exposed only to US, a very limited increase in intracellular ROS production was observed, whereas the sonodynamic treatment of PC-3 cells incubated with the different Chlor formulations induced a significant increase in ROS production (Figure 7). The highest level of intracellular ROS generation was achieved 30 min after the exposure of PC-3 cells to US for all the Chlor formulations tested, being the higher ROS levels obtained with Chlor-Lipo (iMFI ratio 8.2 ± 0.7, *p* < 0.001) and Chlor-PLGA NSs (iMFI ratio 12.3 ± 1.0, *p* < 0.001). These results mirror the ones showing the higher cytotoxicity by the sonodynamic treatment with Chlor-Lipo and Chlor-PLGA NSs compared to the sonodynamic treatment with Chlor or Chlor-SLN (Figure 5).

**Figure 8:**
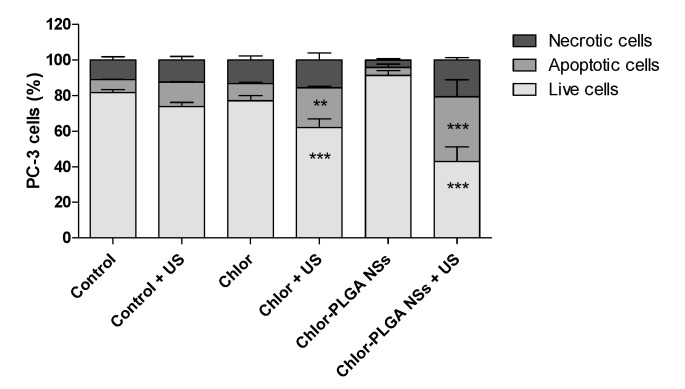
Induction of cell apoptosis on PC-3 cells after sonodynamic treatment with Chlor and Chlor-PLGA NSs. PC-3 cells were exposed to Chlor, Chlor-PLGA NSs (5 µM) for 24 h and then where required underwent to US exposure (1.5 MHz for 5 min at 1.5 W/cm^2^) or left untreated. Apoptosis assays were carried out by flow cytometry, following APC-Annexin V and PI staining. A percentage of apoptotic cells (positive to APC-Annexin V and negative to PI) and necrotic cells (positive to APC-Annexin V and PI) and live cells (negative to APC-Annexin V and PI) was evaluated 24 h after treatment. Statistical significance vs. untreated cells (i.e., control): ** *p* < 0.01, and *** *p* < 0.001.

The effect of sonodynamic stimulation of Chlor formulations was also studied from a cell death point of view. A significant increase in necrotic (15.63 ± 4.01%) and apoptotic (22.39 ± 0.92%) cells was observed when PC-3 cells were exposed to Chlor and treated with US. However, the highest percentage of necrotic and apoptotic cells was obtained when cells were treated with Chlor-PLGA NSs and then exposed to US stimulation (20.63 ± 1.41% and 39.35 ± 9.53%, respectively) (Figure 8).

### 3.5. Effect of the Sonodynamic Treatment with the Chlor formulations on DU-145 Spheroids

**Figure 9:**
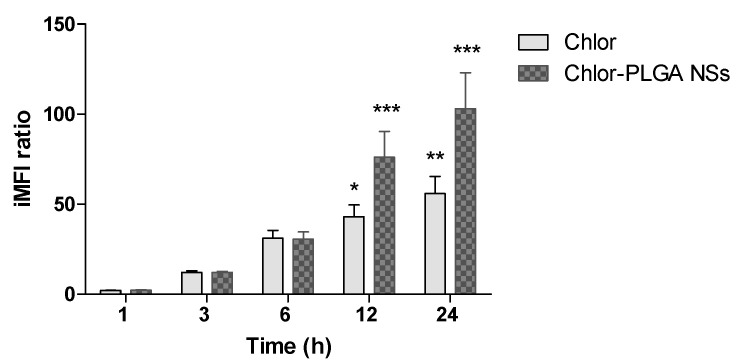
Chlor uptake by DU-145 cells. DU-145 cells were incubated with Chlor and Chlor-PLGA NSs (5 µM) for 1, 3, 6, 12 and 24 h. Fluorescent signal was detected using a flow cytometer at 488 nm excitation to measure the intracellular Chlor and expressed as the iMFI ratio calculated as reported in Materials and Methods. Statistical significance vs. 1 h incubation: * *p* < 0.05; ** *p* < 0.01; *** *p* < 0.001.

Since PC-3 cells were not able to form compact spheroids to be treated with US, we decided to use the human prostate cancer cell line DU-145 to generate spheroids. We decided to investigate on three-dimensional (3D) prostatic cancer cell cultures the sonodynamic activity of free Chlor and of the Chlor formulation with the best sonodynamic effect on two-dimensional (2D) prostatic cancer cell cultures, i.e., Chlor-PLGA NSs. Thus, we first determined the ability of Chlor and Chlor-PLGA NSs to be taken up by DU-145 cells by flow cytometry. A significant time dependent fluorescent signal was observed when cells were exposed for 12 and 24 h to Chlor (iMFI ratio 43.0 ± 6.5, *p* < 0.05 and 55.9 ± 9.5, *p* < 0.01, respectively). However, this fluorescent signal was significantly higher when cells were exposed to Chlor-PLGA NSs compared to free Chlor both at 12 h (iMFI ratio 76.00 ± 14.42 and 43.10 ± 6.53, respectively) and 24 h incubation (iMFI ratio 103.00 ± 20.05 and 55.90 ± 9.58, respectively) (Figure 9).

**Figure 10:**
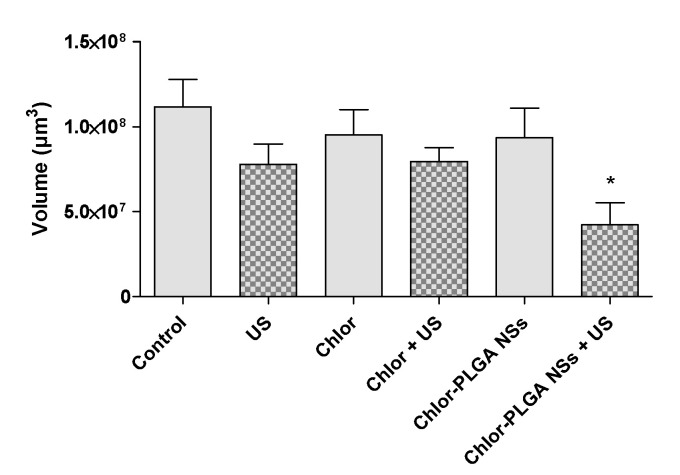
Effect of sonodynamic treatment on DU-145 spheroid volume. DU-145 spheroids were left untreated or treated with Chlor or Chlor-PLGA NSs (10 µM) for 24 h and then, where required, exposed to US (1.5 MHz for 5 min at 1.5 W/cm^2^). Data are expressed as spheroid volume (µm^3^). Statistically significant vs. control: * *p* < 0.05.

Having successfully determined the ability of Chlor-PLGA NSs to induce a significant reduction in cell viability under US exposure (Figure 5), a 3D prostate cancer model was then used to evaluate their efficacy compared to Chlor activity. Indeed, 3D spheroids show a more closely cellular organization to the in vivo tumor features than classic 2D models, becoming, therefore, a helpful tool for investigating drugs and drug delivery systems [39]. Spheroids were treated with free Chlor or Chlor-PLGA NSs and then exposed or not to US. Then, the effect of each treatment was evaluated by determining the volume of each spheroid and also by staining the spheroids with PI to identify necrotic cells. As shown in Figure 10, a significant reduction of spheroid volume was observed when DU-145 spheroids were treated with a combination between Chlor-PLGA NSs and US (*p* < 0.05), while no significant reduction in spheroid volume was observed for the other conditions. In particular, any statistically significant difference in the spheroid volume was observed after the combined treatment with free Chlor and US compared to untreated spheroids.

**Figure 11:**
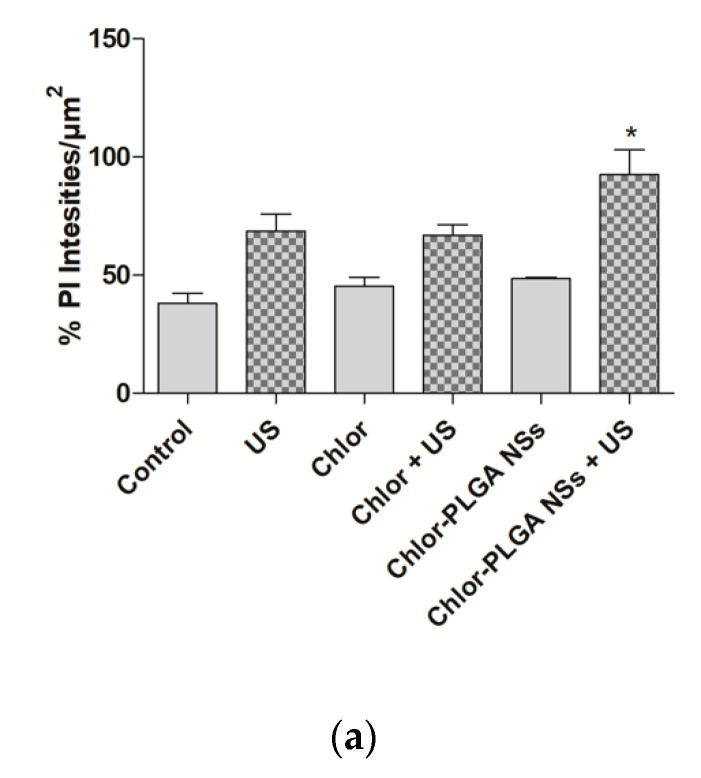

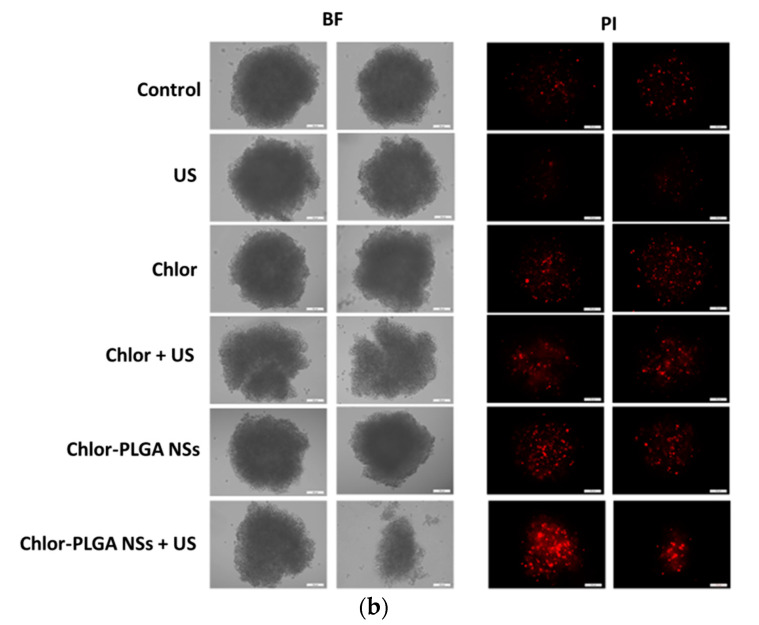
Optical (bright field, BF) and fluorescence (propidium iodide, PI) micrographs of DU-145 spheroids. DU-145 spheroids were left untreated or exposed to Chlor or Chlor-PLGA NSs and their respective combinations with US. Spheroids were stained with PI 24 h following treatment. Panel (**a**) shows PI quantification expressed as % of PI fluorescence intensity/µm^2^. Statistically significant vs. no US exposure: * *p* < 0.05. Panel (**b**) shows representative pictures of spheroids, taken 24 h after each treatment, in bright field (BF) or stained with PI in fluorescence field. Magnification: 10×. Scale bars: 100 μm.

Moreover, PI evaluation identified a slight increase of necrotic cells when DU-145 spheroids were treated with Chlor-PLGA NSs and then exposed to US, observed as well in the quantification of PI signal (Figure 11a,b).

## 4. Discussion

Although natural chlorophylls are well-known nontoxic photosensitizers, easy to restock and widespread, the literature reports only a few studies about their use as sonosensitizing agents [20]. In this work, chlorophyll was derived from the *Galipea longiflora* leaves and used as it is in nature. To counteract its hydrophobicity, which hampers its formulation in aqueous media and distribution in the human body, different nanocarriers (i.e., liposomes, solid lipid nanoparticles and polymer nanoparticles) have also been investigated to identify a proper formulation for the sonodynamic treatment of cancer. Despite the differences in composition and structure, all the Chlor-containing nanocarriers showed a mean diameter in the range of 140–250 nm, with a negative zeta potential and a high EE. All the formulations were stable for at least one month of storage, except for PLGA NCs, which also showed a lower EE if compared to that of NSs; thus, we abandoned this formulation in the further steps of the work. No matter which formulation, incorporated Chlor interacted with the nanocarrier components, lowering the thermogram peak temperature, as observed by DSC analysis. Concerning Chlor release, PLGA NSs showed a slower leakage than that of liposomes and SLN, probably due to a different disposition of Chlor in the matrix system.

To perform in vitro experiments on human prostate cancer PC-3 cells, a non-cytotoxic Chlor concentration was selected (5 µM), being in these conditions the bare nanocarriers non cytotoxic as well. Then, to properly tune the sonodynamic treatment protocol, Chlor intracellular uptake was evaluated on PC-3 cell line and the maximum level was observed after 6 h of incubation for Chlor-Lipo or Chlor-SLN and after 24 h of incubation for Chlor or Chlor-PLGA NSs. These data are in agreement with previous studies on Chlor, in which similar incubation times for cancer cell uptake were observed [40]. Moreover, fluorescence microscopy analysis revealed that Chlor (free or entrapped) has principally a cytoplasmic distribution after the incubation with PC-3 cells.

The sonodynamic treatment with the different Chlor formulations showed a significant inhibition of PC-3 cell growth, contrary to US or Chlor formulations alone. The difference observed in the sonodynamic cytotoxicity of free Chlor when compared to Chlor-loaded nanocarriers might be ascribed to different mechanisms of cell internalization, since it is known that Chlor enters cells by diffusion [41], whereas liposomes and nanoparticles mostly enter cells by endocytosis [42,43]. Noteworthy, the highest decrease in cancer cell growth was obtained with US associated to Chlor-PLGA NSs, even if the level of Chlor cellular internalization after incubation with Chlor-Lipo, Chlor-SLN and Chlor-PLGA NSs was quite the same. By analyzing the physico-chemical characteristics of Chlor nanocarriers, Chlor-PLGA NSs showed the lowest mean diameter and zeta potential values. Indeed, it has been reported in the literature that particle size and aggregation can influence the efficiency of a sonosensitizer loaded into nanoparticles [44]. Moreover, PLGA NSs showed a slower and more controlled release of incorporated Chlor. The highest sonodynamic cytotoxicity observed with Chlor-PLGA NSs can be also related to the interaction of the intracellular energy locally generated by US with the nanoparticle structure [11]. Indeed, recent studies have shown that nanoparticles with rough surfaces can promote a reduction in the acoustic cavitation threshold, positively favoring the effect provoked by US cavitation [45]. PLGA nanoparticles, with their porous surface, seem to confirm this statement, as investigated studying low-intensity US and porous PLGA on peripheral nerves [46]. Therefore, it can be assumed that polymer nanoparticles, such as PLGA ones, loaded with a sonosensitizer, can be considered not only as a tool to increase the sonodynamic activity, but also as an effective vehicle able to target cancer cells and tumor tissues.

To confirm that sonodynamic treatment led to cytotoxic effects through Chlor-mediated intracellular ROS production, ROS levels were determined according to DCFH-DA assay by flow cytometry. A significant increase in ROS production was observed in PC-3 cells after Chlor-PLGA NSs exposure to US, mirroring the cytotoxicity data. The exposure to US alone induced a very limited increase in intracellular ROS, while the highest level was obtained 30 min after US exposure for all the Chlor formulations tested, being the higher ROS levels obtained with Chlor-Lipo and Chlor-PLGA NSs. Furthermore, pre-incubation of PC-3 cells with a ROS scavenger, i.e., NAC, suppressed the cytotoxicity of the sonodynamic treatment with all the Chlor formulations tested, compared to untreated cells, emphasizing the role of ROS in the sonodynamic cytotoxic effect. These data are in agreement with Wang et al. [47] that underline the strong ROS production as the fundamental step in the sonodynamic killing of target cells. Moreover, the US-mediated Chlor activation seems to be selective according to cell type, being effective against human prostatic cancer PC-3 cells but not against human fibroblast HDF 106-05 cells. Experimental evidence suggests that outcome and selectivity of the sonodynamic treatment [48] could be strongly supported by the relevant differences between cancer and non-cancerous cells in cell structure and mechanical properties and then in US responsiveness [49].

It is well known that low ROS levels inside cells play a role of mediating intracellular signals, whereas high ROS levels can lead to cell death as apoptosis and necrosis [50]. In order to evaluate the percentage of apoptosis and necrosis induced by the combined treatment with US and Chlor formulations, Chlor-PLGA NSs, as the formulation with the highest sonodynamic cytotoxicity, and free Chlor as reference were chosen. Chlor-PLGA NSs sonodynamic activation was able to induce a significant increase in apoptotic and necrotic cell death, doubling the values obtained by the sonodynamic activation of free Chlor. These results reinforce the data previously reported, showing that the combined action of US and Chlor-PLGA NSs led to an increase in ROS production, triggering the mechanisms responsible of cell death.

Once demonstrated the capability of Chlor to act as a sonosensitizer on a 2D monolayer cell culture model of human prostate cancer cells, attention shifted to evaluate the sonodynamic treatment effect with US and Chlor (as free drug or loaded into PLGA NSs) on a 3D spheroid culture model. For spheroid development, DU-145 cell line was used, as PC-3 cells showed a poor capacity for cell adhesion and aggregation. Before performing the sonodynamic experiments on DU-145 spheroids, a Chlor cellular uptake evaluation was performed on this cell line; a particularly high Chlor uptake was observed 24 h after the incubation with both free Chlor and Chlor-PLGA NSs. Since spheroids are organized in several layers of cells, an increased non-cytotoxic concentration of Chlor (i.e., 10 μM) compared to that used for 2D cell monolayer (5 μM) was used. After sonodynamic treatment with Chlor-PLGA NSs, a reduction of about 60% in spheroid volume compared to untreated spheroids was observed. It is interesting to note that the volume of spheroids treated only with Chlor-PLGA NSs, therefore not subjected to US exposure, was unaffected. By using PI to evaluate cell death, an increase in PI fluorescence in spheroids treated with US and Chlor-PLGA NSs was also observed, indicating an increased damage of cell membranes caused by the sonodynamic activation of Chlor. If compared to untreated spheroids, the use of Chlor-PLGA NSs respect to free Chlor was more effective in leading to cytotoxicity under US exposure. These data are in accordance with those obtained using 2D monolayer cell cultures.

## 5. Conclusions

This work demonstrates the effectiveness of Chlor as sonosensitizer in killing prostate cancer cells, thanks to ROS generation triggered by Chlor US-mediated activation. The Chlor sonodynamic cytotoxicity was observed in both prostate cancer 2D cell monolayer cultures and 3D spheroids. Moreover, Chlor loading into nanocarriers was able to influence its sonodynamic activity by enhancing the cytotoxic effect, especially using polymer nanoparticles, such as PLGA NSs. Further research should first focus their attention on enhancing the nanoparticle ability to provoke cavitation, i.e., boosting and increasing not only the morphological but also the chemical features of the nanoparticle surface.

## Figures and Tables

**Table 1:** Physico-chemical characteristics of Chlor-loaded nanocarriers (*n* = 3).

Parameters	Chlor-Lipo	Chlor-SLN	Chlor-PLGA NSs	Chlor-PLGA NCs
Mean diameter (nm) ± S.D.	165 ± 2	251 ± 3	140 ± 2	173 ± 4
PDI	0.124	0.133	0.078	0.091
Zeta potential (mV) ± S.D.	−6.48 ± 0.64	−20.12 ± 2.40	−44.40 ± 0.42	−47.92 ± 2.44
EE (%)	98 ± 2	98 ± 2	97 ± 3	87 ± 4
DL (%)	1.8	9.1	4.6	3.6

**Table 2:** DSC analysis of empty and Chlor-loaded nanocarriers.

Formulation	T_onset_ (°C)	T_peak_ (°C)
Empty Lipo	51.6	53.6
Chlor-Lipo	47.8	54.2
Empty SLN	44.5	48.5
Chlor-SLN	42.6	48.0
Empty PLGA NSs	52.3	54.2
Chlor-PLGA NSs	51.7	53.7
